# Supplementary material for: Assessing biomarker trajectories for mortality risk in peritoneal dialysis: A focus on multivariate joint modeling
Source: PLoS One. 2025 Jul 28;20(7):e0320385. doi: 10.1371/journal.pone.0320385 (PMC12303332; doi:10.1371/journal.pone.0320385)
Supplement: S1 File — Analysis code has been included in the supplementary materials. Additionally, all code is freely accessible via GitHub at https://github.com/basolmerve/MultivariateJM_Supplementary. (ZIP) [file pone.0320385.s001.zip › MultivariateJM_Supplementary-main/figure/AUCPlots.pdf]

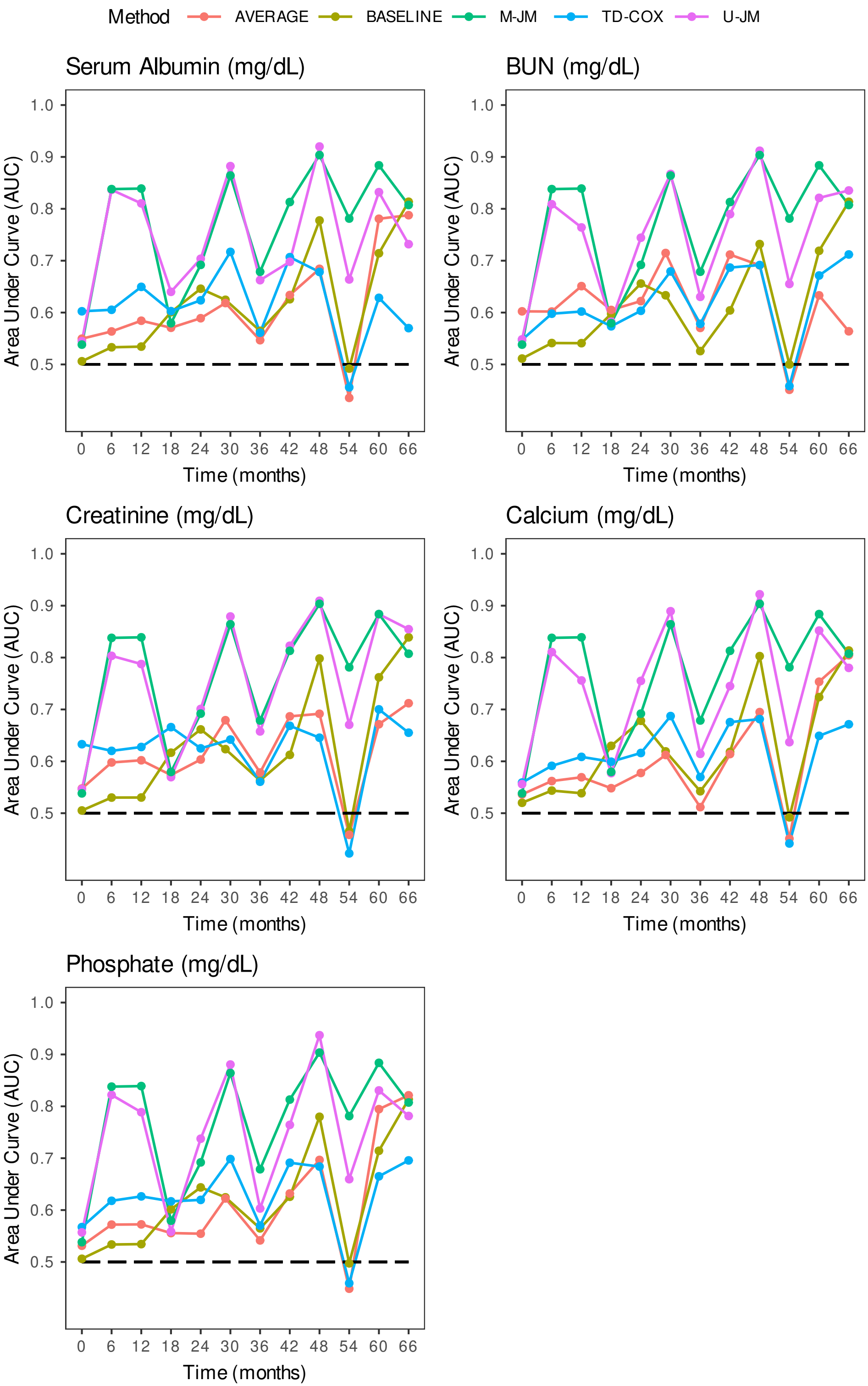

Method
AVERAGE
BASELINE
M-JM
TD-COX
U-JM
Serum Albumin (mg/dL)
BUN (mg/dL)
Creatinine (mg/dL)
Calcium (mg/dL)
Phosphate (mg/dL)
Area Under Curve (AUC)
Time (months)
0.5
0.6
0.7
0.8
0.9
1.0
0
6
12
18
24
30
36
42
48
54
60
66
