# Supplementary figures and images for: Assessing biomarker trajectories for mortality risk in peritoneal dialysis: A focus on multivariate joint modeling
Source: PLoS One. 2025 Jul 28;20(7):e0320385. doi: 10.1371/journal.pone.0320385 (PMC12303332; doi:10.1371/journal.pone.0320385)

**Method** — AVERAGE — BASELINE — M-JM — TD-COX — U-JM

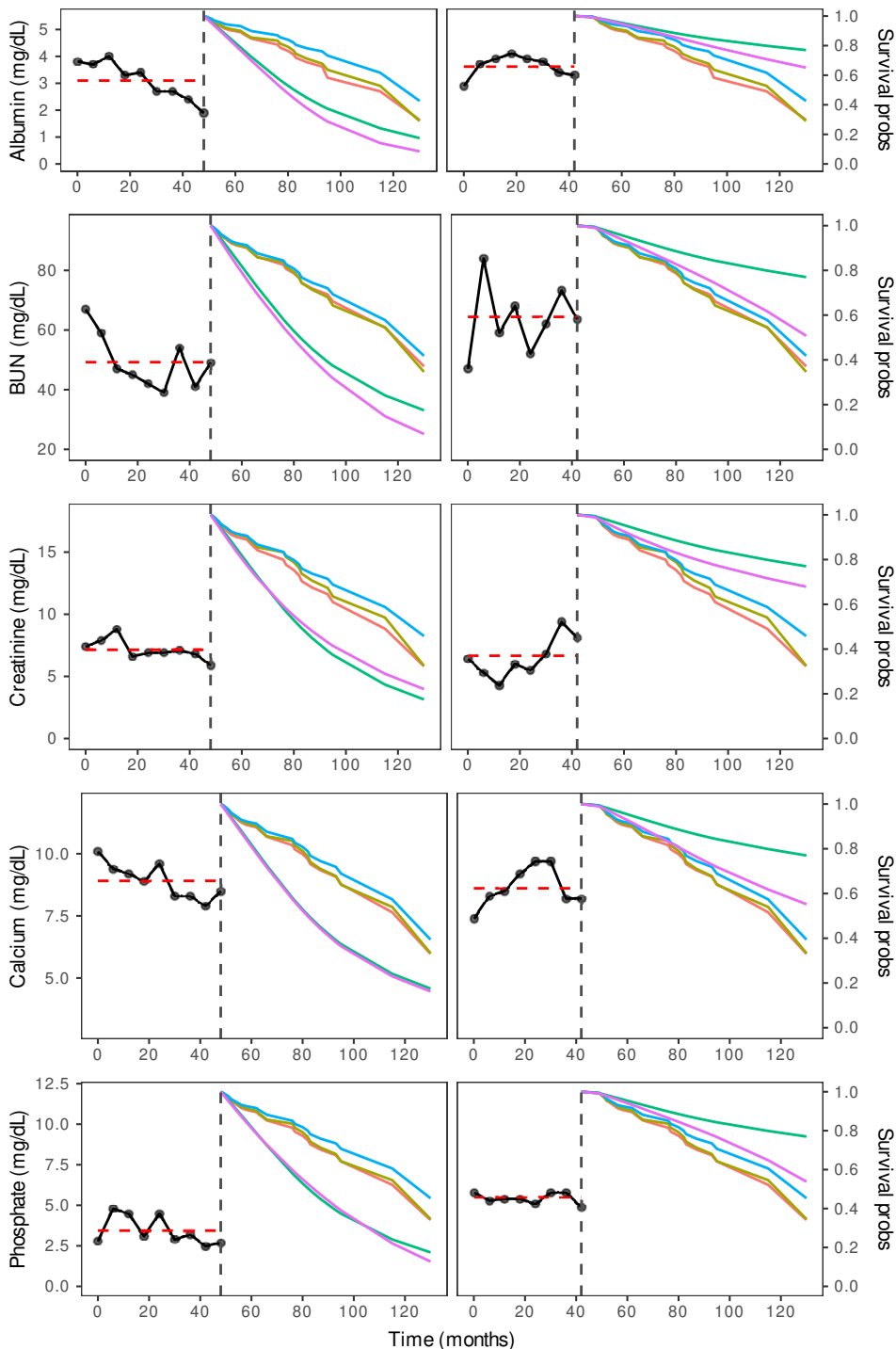

Supplement: S1 File — Analysis code has been included in the supplementary materials. Additionally, all code is freely accessible via GitHub at https://github.com/basolmerve/MultivariateJM_Supplementary. (ZIP) [file pone.0320385.s001.zip › MultivariateJM_Supplementary-main/figure/dynPred.pdf]

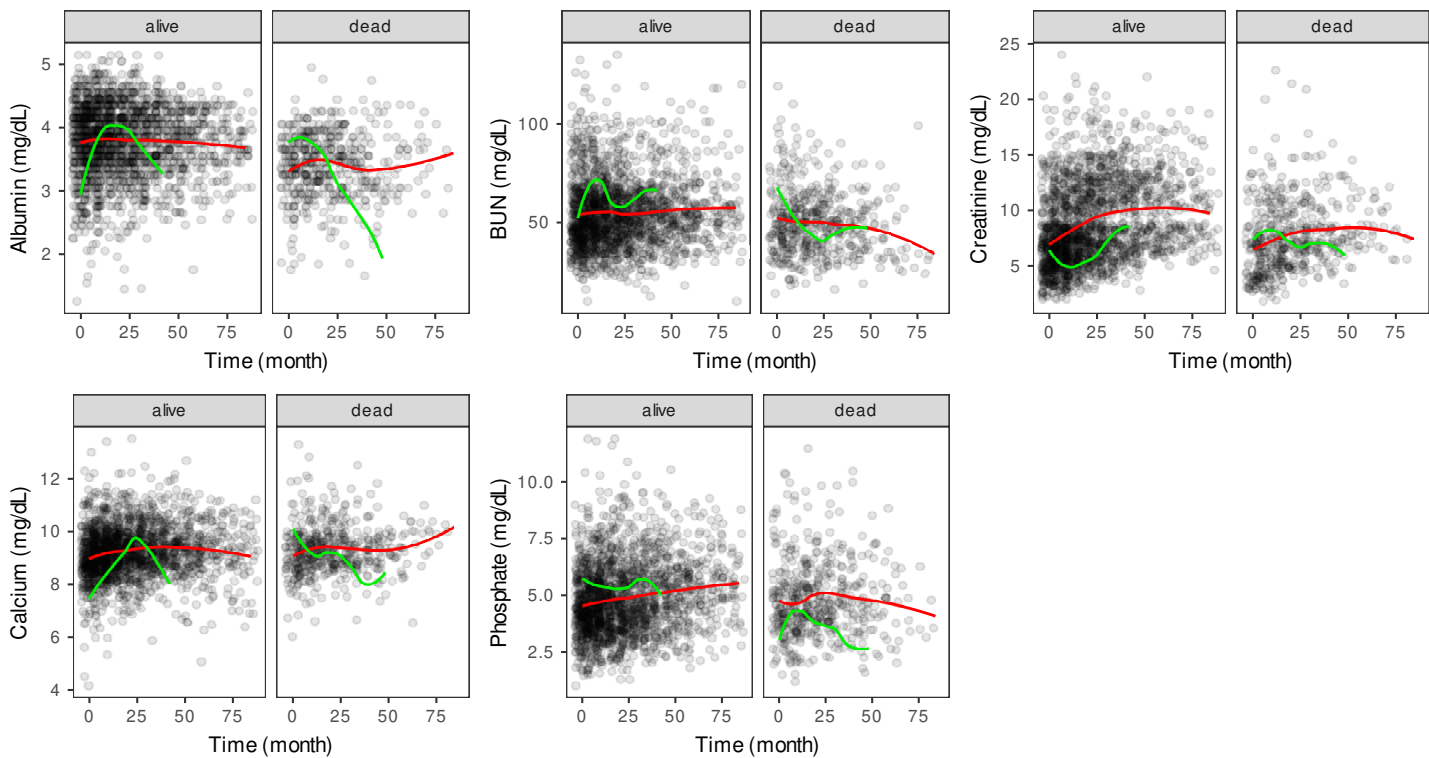

Supplement: S1 File — Analysis code has been included in the supplementary materials. Additionally, all code is freely accessible via GitHub at https://github.com/basolmerve/MultivariateJM_Supplementary. (ZIP) [file pone.0320385.s001.zip › MultivariateJM_Supplementary-main/figure/fig1.pdf]
