# Supplementary material for: Assessing biomarker trajectories for mortality risk in peritoneal dialysis: A focus on multivariate joint modeling
Source: PLoS One. 2025 Jul 28;20(7):e0320385. doi: 10.1371/journal.pone.0320385 (PMC12303332; doi:10.1371/journal.pone.0320385)
Supplement: Attachment — Submitted filename: response_to_reviewers.docx [file pone.0320385.s002.docx]

**Response to Editorial Comments**

Journal requirements:

When submitting your revision, we need you to address these additional requirements.

1. Please ensure that your manuscript meets PLOS ONE's style requirements, including those for file naming. The PLOS ONE style templates can be found at

https://journals.plos.org/plosone/s/file?id=wjVg/PLOSOne_formatting_sample_main_body.pdf and

https://journals.plos.org/plosone/s/file?id=ba62/PLOSOne_formatting_sample_title_authors_affiliations.pdf.

**Response:** Thank you for your comment. We tried our best to follow journal’s style in the revised manuscript.

2. Please note that PLOS ONE has specific guidelines on code sharing for submissions in which author-generated code underpins the findings in the manuscript. In these cases, we expect all author-generated code to be made available without restrictions upon publication of the work. Please review our guidelines at https://journals.plos.org/plosone/s/materials-and-software-sharing#loc-sharing-code and ensure that your code is shared in a way that follows best practice and facilitates reproducibility and reuse.

**Response:** Thank you for your comment. All analysis code used in this study has been provided as supplementary material. In addition, to promote transparency, reproducibility, and ease of reuse, we have made the full set of analysis scripts publicly available via GitHub at the following URL: https://github.com/basolmerve/MultivariateJM_Supplementary.

This information has also been clearly stated in the Data Availability section of the manuscript, in accordance with PLOS ONE’s code sharing policy.

3. In the online submission form, you indicated that [The peritoneal dialysis data used to support the ﬁndings of this study are available from the corresponding author upon request.].

All PLOS journals now require all data underlying the findings described in their manuscript to be freely available to other researchers, either 1. In a public repository, 2. Within the manuscript itself, or 3. Uploaded as supplementary information.

This policy applies to all data except where public deposition would breach compliance with the protocol approved by your research ethics board. If your data cannot be made publicly available for ethical or legal reasons (e.g., public availability would compromise patient privacy), please explain your reasons on resubmission and your exemption request will be escalated for approval.

**Response**: Thank you for your valuable feedback and for highlighting the data availability policy of PLOS journals. We appreciate the importance of ensuring that research data are accessible to the scientific community. However, we must address specific ethical and legal constraints related to the peritoneal dialysis dataset used in this study.

The dataset includes personal information and clinical data of patients who remain under surveillance, subjecting it to the General Data Protection Regulation (GDPR) and other privacy regulations. Publicly sharing this dataset would compromise patient privacy and violate the protocol approved by our research ethics board.

To reconcile the need for data accessibility with these obligations, we propose an alternative approach. The data can be shared with applicant upon request after removing patient-specific identifiers to ensure anonymity, a process that still requires prior permission from our Institutional Executive Board. Researchers interested in accessing the data may contact the data holder (the Department of Internal Medicine, Division of Nephrology, at Erciyes University) or senior author (Murat Hayri Sipahioglu, https://avesis.erciyes.edu.tr/mhsipahioglu), and such requests will be forwarded to the Executive Board for approval.

We therefore respectfully request an exemption from the requirement to deposit the data publicly. Instead, we will include a statement in the manuscript specifying that the data are available from the senior author upon reasonable request, contingent upon approval by the Institutional Executive Board. We think this approach upholds the principles of open science while adhering to our ethical and legal responsibilities.

We included below paragraph under “Data Availability” section:

“The peritoneal dialysis data supporting the findings of this study are available from the Department of Internal Medicine, Division of Nephrology, at Erciyes University (via https://tip.erciyes.edu.tr) upon reasonable request and subject to approval by the Institutional Executive Board, due to ethical and legal restrictions related to patient privacy and General Data Protection Regulation (GDPR) requirements. Researchers may also contact the senior author (Dr. Murat Hayri Sipahioglu, https://avesis.erciyes.edu.tr/mhsipahioglu), who can facilitate the data access process by forwarding requests to the appropriate institutional committee.

We are grateful for your understanding and welcome any further questions or suggestions you may have.

4. We are unable to open your Figure file [fig1.eps, dynPred.eps and AUCPlots.eps]. Please kindly revise as necessary and re-upload.

**Response:** Thank you for your comment, and we apologize for the technical issues you encountered. We have uploaded the original EPS files to the PACE system and additionally converted the corresponding figures to TIFF format, as required. To ensure compatibility, we have submitted all figures in three formats: EPS, TIFF, and PDF. If there are still any issues accessing the figures, please do not hesitate to let us know, and we will be happy to assist further.

**Response to Reviewer’s Comments**

**Reviewer #1:** The authors analyzed mortality risk data with a restrospective cohort of 417 peritoneal dialysis patients followed-up between 1995 and 2016 using both traditional and advanced statistical approaches. They concluded that joint models outperformed other traditional approaches. Also, serum albumin, creatine and phosphorus showed significance as mortality predictors.

We would like to sincerely thank the reviewer for their valuable and constructive comments. Their suggestions have significantly contributed to improving the clarity and overall quality of the manuscript. Each point raised has been carefully addressed to the best of our ability, and detailed explanations of the revisions have been provided in our point-by-point responses. The corresponding changes have been made in the manuscript and are clearly marked using track changes. Line references in our responses refer to the tracked version of the revised manuscript.

1. Inclusion and exclusion criteria. Did the study include any women who pregnant?

**Response:** We did not include any pregnant woman in the study. This criterion is also included in the Exclusion Criteria under Materials and Methods section as “… (iv) pregnancy; ….”. (line 100)

2. Two patients were selected and excluded to serve as the test set. Please provide more in detail information on this design and rational.

**Response:** Thank you for your valuable comment requesting further details on the design and rationale for selecting and excluding two test patients. To ensure an unbiased evaluation, two patients—one censored and one who died—were randomly selected from the cohort without considering their biomarker trajectories (e.g., albumin, creatinine, calcium, phosphorus, and blood urea nitrogen). These patients were excluded from the training dataset (n=415) to serve as an independent test set, allowing assessment of model performance on unseen data. Unlike traditional survival models, such as Cox regression, which derive population-based predictions, our multivariate joint modeling approach estimates patient-specific survival probabilities through random effects, capturing individual biomarker dynamics. Consequently, evaluating predictive performance at the patient level, as demonstrated with two representative patients, was sufficient to illustrate the models’ capabilities, and a larger test set was unnecessary for this purpose. This design leverages the strength of dynamic, personalized predictions, aligning with our study’s objectives. We have included a concise explanation of this approach in the “Study Design and Participants” section (lines 103 – 109).

3. Continuous variables were summarized depending on the data distribution. Please be more specific.

**Response:** We selected appropriate summary statistics depending on the normality of continuous variables. To clarify this, we added/modified below text to the “Statistical Analysis” section (lines 142 – 145).

“The normality of continuous variables was assessed using the Shapiro-Wilk test and graphical methods such as histograms, Q-Q plots, and box plots. Normally distributed variables were summarized using means and standard deviations while nonnormally distributed variables were summarized using medians and minimum and maximum values.”

4. Please clarify whether the proportional hazards assumptions were evaluated. What if the assumptions were not satisfied.

**Response:** Thank you for highlighting this important aspect of survival analysis. We evaluated the proportional hazards (PH) assumption for all fitted survival models using standard diagnostic tests in R programming language (i.e., “cox.zph” function under survival package). All survival models included a consistent set of covariates: age, body mass index (BMI), history of hemodialysis (preHD), total number of renal and comorbid diseases (TCRD), peritoneal membrane transport characteristic (TC), and one of the selected biomarkers—albumin, creatinine, blood urea nitrogen (BUN), phosphorus, or calcium. The results indicated that the PH assumption was not violated globally across the models. However, we observed that the TC covariate exhibited a local violation of the PH assumption in some models. Despite this, it did not compromise the overall model validity in global tests. Given the clinical relevance of TC in the progression of PD outcomes, we retained it in the survival models. Moreover, we are aware that conducting multiple tests for the PH assumption—one for each covariate in each fitted model—can increase the risk of Type I error. For this reason, we based our modeling decisions on the overall pattern of test results rather than isolated violations. In summary, the PH assumption was generally satisfied across the survival models, and no violations were observed that would compromise the validity or interpretability of the joint models used in downstream analyses.

We did not include the detailed test results in the manuscript, as presenting them in a single table would have made the content overly long and difficult to interpret. However, we summarized this information in the Results section (lines 240 – 245).

5. It’s unclear how M2 (average value) were conduct in a Cox PH model as the values have been averaged.

**Response:** Thank you for your comment. The M2 model is structurally similar to the Cox PH model that incorporates baseline biomarker values (i.e., M1); however, instead of using baseline values, it includes the arithmetic mean of the repeatedly measured biomarkers across the follow-up period. While M2 does not account for the time-dependent trajectory of biomarkers, it captures their average effect over time, offering a simpler alternative for prediction modeling.

To implement M2, we calculated the average value of each longitudinal biomarker for each patient and included these as covariates in the Cox PH model. This approach, although limited in capturing dynamic changes, is commonly used for its interpretability and simplicity. To improve clarity, we have provided detailed information regarding the mathematical formulation and model specifications in the Appendix section of the manuscript (Appendix A and Table 3).

6. In equation 1, many abbreviations have been used without explanation, except BUN.

**Response:** Thank you for your observation. All abbreviations used in the equations are introduced in the main text upon their first appearance. To maintain readability and avoid interrupting the narrative flow, we have provided the mathematical background and detailed model specifications in the Appendix A section. Additionally, a comprehensive list of abbreviations used in the equations is included as a table in Appendix B, where each term is defined in full for reference.

7. Why wasn’t Age adjusted as a random effect in CR, CA, and PH analysis?

**Response:** Thank you for drawing attention to this issue. There was a typographical error in the model formulations. The Age variable was not included as a random effect in any of the models. However, we noticed that Age was mistakenly listed alongside the random intercept terms in the ALB and BUN models. We have corrected the model equations accordingly (equation 5 between lines 788 and 789).

To improve clarity and avoid overcomplicating the main text, we have provided all theoretical details and complete model specifications in Appendix A and Appendix B of the manuscript.

8. It’s nice to see a clear pattern that joint modeling predicted the survival rate more aligned to the two selected individuals based on Figure 2. However, how do we know whether this is more due to the chance. For example, in Figure 1, the trajectories from two selected individuals looked quite different from two selected individuals.

**Response:** Thank you for the insightful comment regarding the performance of our multivariate joint modeling approach in predicting survival rates for the two selected individuals in Figure 2. We acknowledge the concern about whether the observed patterns are due to chance, particularly given the differing trajectories in Figure 1.

The two test patients were randomly selected without prior consideration of their biomarker trajectories (e.g., albumin, creatinine), ensuring an unbiased evaluation. Our dataset includes a small minority of outlier patients whose biomarker trends deviate from clinical expectations, such as those with improving biomarkers who died or deteriorating biomarkers who survived. For such outliers, our model predictably assigns higher survival probabilities to patients with worsening trajectories and higher probabilities to those with improving trends, consistent with what the models have learnt from complete data. This does not indicate poor model performance but reflects the expected limitations in handling rare outliers, which we confirmed some are prevalent in our dataset.

We did not specifically focus on these outliers during model evaluation but ensured their inclusion during training to evaluate the model’s robustness to real-world variability. While selecting a different pair of test patients could yield varying predictions, potentially with weaker performance for specific cases. However, we think that this would not generalize to the entire population, as our model demonstrates strong predictive performance at patients’ level. The distinct trajectories of individual patients underscore the need for personalized dynamic predictions, as our random-effects multivariate joint model provides, rather than relying on population-based models. This highlights the strength of our approach in capturing patient-specific biomarker dynamics for tailored risk assessment.

9. The conclusion on the method comparison based on one data without simulation is less convincing without some rigorous simulation studies.

**Response:** Thank you for your valuable comment regarding the need for rigorous simulation studies to strengthen our method comparison. Initially, we considered conducting a comprehensive simulation study to enhance the generalizability of our findings. However, the models compared in our study—each with distinct distributional assumptions, particularly in formulating the baseline hazard—posed significant challenges. Specifically, the data generation process would inherently favor the model whose assumptions align with the simulated data’s underlying properties, potentially biasing the comparison. Moreover, the models involve numerous parameters, leading to risks of overparameterization and computational intractability. These complexities, combined with the time-intensive nature of designing a robust simulation framework, prevented us from including such a study. We acknowledge this as a limitation and have added it to the manuscript’s discussion, noting that future research should explore simulation-based validations to further substantiate our findings. Our current comparison, grounded in real-world data, provides a practical foundation for assessing model performance, but we recognize the value of simulations for broader generalization. Below text is added to the limitations of study, within the last paragraph of Discussion section (lines 489 – 495).

“Second, the absence of a simulation study limits our findings’ generalizability. Conducting such a study was impractical due to differing model distributional assumptions, risking biased data favoring specific models, with overparameterization and computational issues. Under favorable conditions, a comprehensive simulation study can be conducted, for instance, by varying sample sizes, predictors, multivariate responses, and baseline hazard specifications, to enhance applicability. We leave this as a further research topic.”

**Reviewer #2:** The authors present a retrospective study investigating mortality risk prediction in PD patients through longitudinal biomarker analysis, comparing traditional and advanced statistical approaches. They showed a highest predictive accuracy of multivariate joint modeling.

I suggest the following revisions:

We would like to sincerely thank the reviewer for their valuable and constructive comments. Their suggestions have significantly contributed to improving the clarity and overall quality of the manuscript. Each point raised has been carefully addressed to the best of our ability, and detailed explanations of the revisions have been provided in our point-by-point responses. The corresponding changes have been made in the manuscript and are clearly marked using track changes. Line references in our responses refer to the tracked version of the revised manuscript.

1. Rationale should be strengthened – for example, how about the current progression analysis of PD patients, including prognostic models and predictors? The use of dynamic prediction models in other chronic disease management? And are there any examples of actual benefits brought by the evaluation of biomarkers trajectories in clinical actions?

**Response:** We have revised the introduction section around this comment. Furthermore, we added a paragraph into discussion section to support revised version of the Introduction section.

Corrections in introduction section correspond to lines 25 – 27, 43 – 49, 51 – 57, 60 – 62, and 68 – 80. Relevant literature also included in the references.

Corrections in Discussion section correspond to line 196 – 300 and 472 – 482.

2. Please explain the reason why patients with survival of fewer than 90 days after starting PD were excluded, and whether any patients with other fatal diseases such as malignant tumor or severe cardiovascular disease were excluded?

**Response:** The exclusion of patients with survival of fewer than 90 days after starting peritoneal dialysis (PD) was based on the need to ensure that deaths could be reasonably attributed to PD-related factors. In clinical studies, a minimum exposure period, typically 90 days, is commonly adopted to establish a meaningful association between PD treatment and mortality outcomes. This threshold helps to distinguish PD-related mortality from deaths due to other acute or pre-existing conditions. Regarding patients with other fatal diseases, such as malignant tumors or severe cardiovascular disease, our clinical practice generally avoids initiating PD in individuals with malignancies or conditions associated with short life expectancy, unless there are no contraindications. As this was a retrospective study, such patients were unlikely to have been included in the cohort, aligning with our standard clinical decision-making principles.

We included above exclusion criterion in the “Exclusion Criteria” under Material and Methods section such as “… (v) existence of other fatal diseases associated with short life expectancy (e.g., malignant tumor, severe cardiovascular disease, etc.);” (lines 100 – 101).

We revised the exclusion criterion about 90 days threshold as “(iii) survival of fewer than 90 days after starting PD since this threshold is typically a minimum exposure period to establish a meaningful association between PD treatment and mortality outcomes;”. Furthermore, related citations included to support our claim (lines 98 – 99).

3. The Charlson Comorbidity Index should be used in the statistical analyses to describe the overall comorbidity.

**Response:** We appreciate the reviewer’s valuable suggestion to incorporate the Charlson Comorbidity Index (CCI) into our modeling process. Unfortunately, we were unable to adjust our models for CCI due to missing data for at least one required variable in the majority of subjects, which prevented accurate computation of CCI scores. To address this, we included a composite variable, the "total number of comorbid and renal diseases (TCRD)," as a proxy for comorbidity burden. While we acknowledge that TCRD does not fully capture the nuanced impact of CCI, it partially accounts for the effects of renal and comorbid conditions, which are components of the CCI. We have noted the inability to use CCI as a limitation in our study, and we believe TCRD provides a reasonable alternative given the data constraints (lines 483 – 489).

4. It is not clear from the methodology section how the authors defined “TCRD” or “number of illnesses”?

**Response:** Thank you for your comment. The variable “TCRD” (total count of comorbid and renal diseases), also referred to as the “number of illnesses,” was defined as the sum of predefined comorbid and renal conditions observed in each PD patient. Specifically, TCRD includes the concurrent presence of diabetes mellitus, coronary artery disease, chronic heart failure, cerebrovascular disease, lung disease, liver disease (i.e., hepatitis), hypertension, polycystic kidney disease, and glomerulonephritis, resulting in a composite score ranging from 0 to 9.

We have clarified this definition in the manuscript under the Clinical Outcome subsection to ensure transparency and reproducibility of the methodology (lines 121 – 127).

5. Please show the precise results of Cox PH regression models for the time-to-event analysis (as mentioned in line 152, page 5 and line 153-155 in page7), and it’s not clear how the “TC” were employed into the model as a variable.

**Response:** Thank you for your observation. Detailed results and parameter estimate of all fitted models, including the Cox proportional hazards and linear mixed-effects (LME) models, are provided in tabular format in Appendix B. Due to space constraints and to improve readability, only the results related to the primary biomarkers of interest (albumin, creatinine, calcium, phosphorus, and BUN) were included in the main Results section.

Additionally, we identified a typographical error in the manuscript. While the variable "peritonitis rate" was found to be statistically significant in the survival model, it was mistakenly referred to as "TC" in the text. This has been corrected, and the appropriate reference to "peritonitis rate" has been inserted in the relevant section (line 233).

6. Please clarify the results of M1-M3 in table2, whether it’s univariate model or multivariate model.

**Repsonse:** Thank you for your question. Models M1 through M4 are univariate, each evaluating the effect of a single biomarker on survival outcomes. In contrast, M5 is a multivariate joint model that simultaneously incorporates all selected biomarkers while accounting for their interrelationships.

We have clarified this distinction in the Statistical Analysis section (lines 163 – 193) and also provided detailed specifications for each model in a summary table within the Appendix (table 3, between lines 722 and 723).

7. It’s confused how the two patients (i.e. test samples) were chosen from the complete dataset? Have they been selected randomly?

**Response:** The two test patients were randomly selected without prior consideration of their biomarker trajectories (e.g., albumin, creatinine), ensuring an unbiased evaluation. We have included below paragraph in the “Study design and participants” section (lines 103 – 111).

“Additionally, to assess model performances, two test patients --one censored and one who died-- were randomly selected without prior consideration of their biomarker trajectories (e.g., albumin, creatinine, etc.), ensuring an unbiased evaluation. Selecting additional patients was unnecessary, as dynamic survival models generate patient-specific predictions by fitting at the individual level. Dynamic survival probabilities were estimated for these patients using each model evaluated in the study. A training dataset of 415 patients was used to develop and fit all survival models, providing a robust basis for comparing predictions.”

8. Please show the study limitations, which is essential for the study generalization.

**Response:** In the last paragraph, before the Conclusion section, we discussed the limitations of our study (lines 483 – 495).

**Revisions made independently of reviewer and editor comments:**

In addition to the revisions made in response to the reviewers’ and editor’s comments, we also implemented several improvements throughout the manuscript to enhance its clarity, accuracy, and structure. These changes are outlined below:

#1 – Typographical errors were corrected throughout the manuscript.

#2 – Mathematical notations originally presented in the Statistical Analysis section were moved to the Appendix, where both the theoretical formulations and model outputs are described in detail.

#3 – In-text descriptions of the statistical modeling approaches (M1 to M5) were added to the Statistical Analysis section. Detailed formulations and specifications were moved to the Appendix as noted in #2.

#4 – Several typographical errors in Table 1 were corrected (e.g., “phosphate” was revised to “phosphorus”), including errors in the table footnote.

#5 – In Table 2, the estimate for the CA biomarker in Model M4 was mistakenly reported as a reciprocal value and has been corrected.

#6 – The Data Availability section was revised and expanded to include detailed information regarding access to the analysis code.

#7 – References were updated and added where appropriate to support the revised content of the manuscript.

#8 – Second affiliation, i.e., Dept. of Biostatistics, Sakarya University, is added to the corresponding author (Dr. Dincer Goksuluk).
